# Socially-marketed rapid diagnostic tests and ACT in the private sector: ten years of experience in Cambodia

**DOI:** 10.1186/1475-2875-10-243

**Published:** 2011-08-18

**Authors:** Shunmay Yeung, Edith Patouillard, Henrietta Allen, Duong Socheat

**Affiliations:** 1 Department of Global Health & Development, Faculty of Public Health & Policy, London School of Hygiene & Tropical Medicine, 15-17 Tavistock Place, London WC1H 9SH, UK; 2 Population Services International, No. 29, 334 Street, Boeung Keng Kang 1, Khan Chamcar Mon, Phnom Penh, Cambodia; 3 National Center for Parasitology, Entomology and Malaria Control, No 372, Preah Monivong Boulevard, Phnom Penh, Cambodia

## Abstract

Whilst some populations have recently experienced dramatic declines in malaria, the majority of those most at risk of *Plasmodium falciparum *malaria still lack access to effective treatment with artemisinin combination therapy (ACT) and others are already facing parasites resistant to artemisinins.

In this context, there is a crucial need to improve both access to and targeting of ACT through greater availability of good quality ACT and parasitological diagnosis. This is an issue of increasing urgency notably in the private commercial sector, which, in many countries, plays an important role in the provision of malaria treatment. The Affordable Medicines Facility for malaria (AMFm) is a recent initiative that aims to increase the provision of affordable ACT in public, private and NGO sectors through a manufacturer-level subsidy. However, to date, there is little documented experience in the programmatic implementation of subsidized ACT in the private sector. Cambodia is in the unique position of having more than 10 years of experience not only in implementing subsidized ACT, but also rapid diagnostic tests (RDT) as part of a nationwide social marketing programme. The programme includes behaviour change communication and the training of private providers as well as the sale and distribution of Malarine, the recommended ACT, and Malacheck, the RDT. This paper describes and evaluates this experience by drawing on the results of household and provider surveys conducted since the start of the programme.

The available evidence suggests that providers' and consumers' awareness of Malarine increased rapidly, but that of Malacheck much less so. In addition, improvements in ACT and RDT availability and uptake were relatively slow, particularly in more remote areas.

The lack of standardization in the survey methods and the gaps in the data highlight the importance of establishing a clear system for monitoring and evaluation for similar initiatives. Despite these limitations, a number of important lessons can still be learnt. These include the importance of a comprehensive communications strategy and of a sustained and reliable supply of products, with attention to the geographical reach of both. Other important challenges relate to the difficulty in incentivising providers and consumers not only to choose the recommended drug, but to precede this with a confirmatory blood test and ensure that providers adhere to the test results and patients to the treatment regime. In Cambodia, this is particularly complicated due to problems inherent to the drug itself and the emergence of artemisinin resistance.

## Background

The importance of the private sector in the provision of malaria treatment is widely recognized [1-10]. It is however only recently that its potential role in increasing community access to good quality malaria treatment, through artemisinin combination therapy (ACT) and potentially parasitological diagnosis has been considered. One response has been the Affordable Medicines Facility for malaria (AMFm) initiative, the first phase of which is being rolled out in eight countries. The AMFm aims to increase access to ACT by lowering the cost of the drugs through a manufacturer-level subsidy. In the selected countries, public, private and not-for-profit first-line buyers will be able to purchase nationally-recommended ACT from manufacturers at around US$0.05 per dose, a fraction of their actual cost. In the private sector, it is anticipated that the saving will pass through the distribution chain, so that consumers will be able to purchase ACT at a price comparable to that of older less effective monotherapies (i.e. approximately $0.20 for an adult course) [11]. To date, although there has been a number of pilot studies [12-14], there is little documentation on subsidizing ACT at a programmatic level on which to base this roll-out [13-15].

There is even less information about subsidizing parasitological diagnosis using Rapid Diagnostic Tests (RDTs) in the private sector. This issue has recently come to the fore as a number of countries report dramatic reductions in malaria, and the importance of targeting ACT to those with confirmed malaria has become more apparent. This is reflected in the recently published World Health Organization (WHO) malaria treatment guidelines which now recommend parasitological confirmation prior treatment [16]. The availability of easy-to-use and quality-assured RDTs for malaria [17] means that parasitological confirmation is no longer limited to public health facilities with the capacity to perform microscopy, but can also potentially be offered by community health workers and private providers.

Cambodia is in the unique position of having had a nationwide social marketing programme for both RDTs and ACT in the private sector since 2002. As other countries prepare for the AMFm pilot, much could be learnt from the Cambodian experience. However to date the amount of information available has been limited[15]. The aim of this paper is therefore to describe the development and implementation of Cambodia's social marketing programme and to review the available evidence on the evolution of the programme's outcomes in terms of product awareness, availability, access, cost and quality.

### Study population

**Figure 1:**
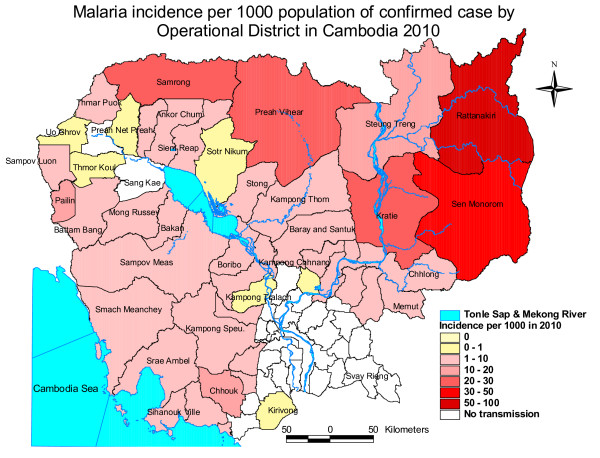
**Malaria Incidence by number of treated cases per 1000 population by operational district, Cambodia 2009 (Source: Dr Siv Sovannaroath, CNM)**.

Cambodia has a population of 13.7 million inhabitants, of which 80% are rural [18] and 40% live with less than US$1.25 per day [19]. Malaria transmission is micro-heterogenous, but generally limited to the relatively sparsely populated hilly and forested areas mainly around the country borders (Figure 1). An estimated three million people are at risk of malaria, of which 1.6 million are considered at high risk because they live within 1 km of the forest, where the local vectors breed [20]. Unlike in much of Africa, it is adults, who work and stay overnight in the forest, who carry the highest burden of the disease. Based on official statistics there has been a downward trend in morbidity in the last decade, with 83,777 outpatient and 4,045 inpatient cases reported in 2009. Overall, malaria is reported to account for 0.6% of outpatient cases and 3.5% of inpatient cases [21]. However, as around 80% of medical treatments are obtained in the private sector [22,23], information from the official Health Information System significantly underestimates the true burden of disease.

The epidemiology of malaria differs remarkably across the country. In the Northeast, malaria transmission is relatively high, with reported annual incidence of 11 to 50 per 1,000 habitants [24], *Plasmodium falciparum *predominates and remains relatively drug-sensitive. By contrast, along the Western border with Thailand, which is known as the epicenter of multi-drug resistant *P. falciparum *malaria [25,26], transmission is generally lower and *Plasmodium vivax *predominates in some parts.

In 2000, Cambodia became the first country to switch its first-line national malaria treatment policy for *P. falciparum *malaria to an ACT of artesunate and mefloquine (ASMQ) [27]. The policy change was accompanied by a number of innovative strategies aimed at increasing free or affordable access to early diagnosis and good-quality treatment and decreasing the use of inappropriate, sub-standard and fake drugs. Artesunate and mefloquine were co-blistered into age-specific packs named "A+M4", "A+M3" and "A+M2" for provision in public health facilities. Parasitological diagnosis was promoted through the introduction of RDTs and the strengthening of capacity for skilled microscopy. In addition, a cadre of community health workers known as Village Malaria Workers (VMWs) were trained to provide free diagnosis and treatment with RDTs and ACT in the most remote and at-risk communities [28,29]. Finally, in recognition of the importance of the private sector, a "social marketing" programme of subsidized co-blistered ASMQ and RDTs was initiated in order to improve the availability and quality of malaria diagnosis and treatment in this sector [30,31]. Social marketing is a relatively common approach for increasing coverage of health services and products in resource limited settings using the tools and concepts of commercial marketing, including promotional activities, branding, pre-packaging and subsidy of public health commodities [32,33].

### Case description: social marketing of RDTs and ACT

#### The development and pilot phase (1999-2001)

**Figure 2:**
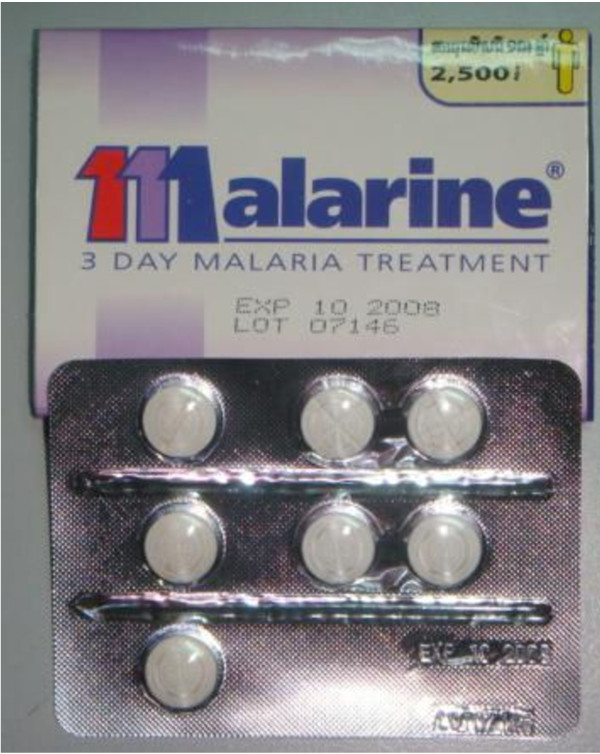
**Malarine for Adult (2008)**.

The social marketing programme was initiated by the European Commission Cambodia Malaria Control Project (EC-CMCP) in partnership with the National Centre for Parasitology, Entomology, and Malaria Control (CNM) and the WHO. The programme started in 1999 with a "pre-pilot" study during which packaging and promotional materials, guidelines for community health providers, wholesale and retail price levels and methods for monitoring and evaluations were developed and pre-tested in one community. The brand name Malarine was chosen for the ACT, with the "M" in Malarine designed to look like three "1's" in order to encourage adherence to the once daily 3-day regime (Figure 2). The drugs, artesunate and mefloquine were procured by the programme. Co-blistering and packaging was undertaken locally in a specially renovated blistering packaging facility with the support of the WHO. For diagnosis, Paracheck^®^, the Histidine Rich Protein 2-based *P. falciparum*-specific RDT used in the public sector, was branded as Malacheck for sale in the private sector.

In 2000, a pilot study was conducted in two districts, one in Battambang province in the Northwest of the country and the other in Kampot province in the South of the country. The pilot involved six wholesalers and 30 health providers [34] and, in addition to the provision of pre-packaged ACT and RDTs, a comprehensive programme of Information, Education and Communication (IEC), training and support to both private drug providers and public health workers was implemented. An evaluation of the pilot found that that the *"observed result [was] promising" *and resulted in plans to scale-up [35].

### Scale up and handover to Population Services International (2002-2003)

In March 2002, the nationwide social marketing project was officially launched, targeting the 17 malaria endemic provinces. A single company was contracted to handle the distribution of the products and an advertising agency was used to develop a nationwide promotional campaign. Then followed a transitional period with the EC-CMCP closing at the end of the year, and the management of the programme being handed over to Population Services International (PSI) in February 2003.

### The Nationwide Social Marketing Programme (2004 to present)

Since handover, PSI have continued to run the programme and are responsible for all aspects including procurement, packaging, sales and distribution of Malarine and Malacheck; training of private providers; and behaviour change communications. Financial support is provided by the Global Fund to Fight AIDS, Tuberculosis and Malaria (GFATM).

### The products

Until 2007, artesunate and mefloquine were procured by PSI from Mepha Pharma Ltd (for mefloquine 250 mg) and Guilin Pharmaceutical Co. Ltd (for artesunate 250 mg and 50 mg), and were then locally co-blistered and packaged in two age-specific packs: "Adult" for ages over 13 years (weight > 30 kg) and "Child" for ages 6-13 years (weight 15-30 kg). The local co-blistering and packaging arrangements became increasingly problematic due to issues related to GFATM procurement procedures and the ambiguity over whether the local packaging facility had achieved the necessary "Good Manufacturing Practice" status to allow procurement using GFATM funds. Delays have also been compounded by the repeatedly dashed expectations of the country being able to switch to an effective co-formulated single tablet combination ACT, such as dihydroartemisinin-piperaquine. In both public and private sectors, these problems resulted in severe procurement delays and stock-outs [36] with packaging finally being contracted out to Cipla Ltd in 2009. At the same time, in line with changes in the national malaria treatment guidelines, the dosing of Malarine for Adult was increased and the age banding changed from over 13 years to over 15 years, and an additional adolescent-age pack was introduced for ages 11 to 15 years.

As for the RDT, this continued to be the Paracheck^® ^product, branded as "Malacheck" until 2009-2010. Since then, in line with changes in the national guidelines, the RDT was switched to a "combination" HRP2/pLDH RDT that diagnoses both *P. falciparum *and non-*P. falciparum *malaria, and is still marketed under the brand name Malacheck.

### Distribution

Malarine and Malacheck are stored initially in PSI's warehouse in Phnom Penh. Stocks for around two months are then distributed to three regional depots from where PSI sales representatives get their supply and distribute to approximately 350 providers per month, mostly located in provincial and district towns. Larger outlets then act as de-facto wholesalers and serve less accessible providers who may also buy from one another [37].

### Cost and pricing

Since the beginning, there has been a recommended retail price (RRP) at which retailers were supposed to sell the products to the end-user. For Malarine (but not Malacheck), the RRP has been printed on the boxes of Malarine, for increased consumer awareness and to encourage provider adherence. Under the EC-CMCP pilot, the RRP for Malarine Adult was 7,900 riel (US$1.93), for Malarine Child 4,900 riel ($1.20), and for Malacheck 1,000 riel (US$0.24). After handover, PSI conducted a willingness-to-pay survey and as a result reduced the RRP of Malarine to 2,500 riel (US$ 0.61) for both the adult and child packages [38]. In 2009, the changes in the dosing regimen, age-banding and packaging were accompanied by a halving of the RRP of the "Child" dose to 1,200 riel (US$0.29) and the pricing of the new "Adolescent" dose being set at 1,700 Riel (US$0.41), with no change to the RRP for the "Adult" dose.

Prior to 2009, the cost to PSI of procuring Malarine was US$ 3.11 for the adult blister and US$2.10 for the child blister, including the costs of the drugs, packaging, sampling and testing, freight, packing, insurance, and delivery to the PSI warehouse. In 2009, the changes in dosing and in the packaging facility were associated with an increase in procurement prices to US$3.63 for the adult dose, US$2.34 for the adolescent dose and $1.82 for the child dose (personal communication, PSI Cambodia procurement department). For the RDTs, the procurement cost of the *P. falciparum *specific RDT was around US$ 0.45 per test compared to around $0.59 for the combination LDH/HRP2 test (personal communication, PSI Cambodia procurement department).

Until 2009, in order to allow for a minimum distribution margin of US$0.15 or around 33% per pack, PSI had a differential pricing policy at which they sold products to wholesalers compared to retailers. For wholesalers, Malarine Adult was sold at US$5.00 for a dispenser of 12 packs (equivalent to US$ 0.42 per pack) compared to US$5.50 (or US$ 0.46 per pack) direct to retailers. In 2009, this was replaced by a single wholesale price. Thus, the price for 12-pack dispensers of Malarine for Adult, Adolescent and Child were set at US$5.00 (US$0.42 per pack), US$3.20 (US$ 0.26 per pack) and US$2.20 (US$0.18 per pack) respectively. For Malacheck, PSI originally sold boxes of 10 tests at a price of US$2.20 (US$0.22 per test). With an RRP of US$ 0.24 per test this allowed for a distribution margin of 9%. In 2009, in order to encourage providers to always test before treatment, the wholesale price was dropped to only US$0.50 for 10 tests (US$ 0.05 per test), allowing retailers to increase their margin per test dramatically, from 9% to 380% (Additional File 1).

### Behaviour change communications

Behaviour change communication (BCC) has been a key component on the social marketing strategy since its start and has evolved over time. The aim of the programme is to influence the behaviour of providers and consumers by developing and delivering key messages through a range of media. PSI put an emphasis on using evidence to develop key messages and to evaluate the effectiveness of the BCC programme. The range of media includes mass media advertisements through television and radio spots, community educational activities through mobile video units, distribution of point-of-sale materials such as posters and job aids, training of providers and a "medical detailing" programme. Private providers are trained on malaria diagnosis and treatment during one-day group sessions. Treatment providers are also visited by medically or pharmacy trained " detailers" who provide additional advice and support.

In the early years, the BCC materials focused on promoting Malarine with increasing emphasis being placed on diagnosis in later years. The strategy differs between providers and consumers with Malacheck actively being promoted to providers, whilst parasitological diagnosis (including microscopy) in general rather than Malacheck specifically, is promoted amongst consumers.

### Sales of Malarine and Malacheck

**Figure 3:**
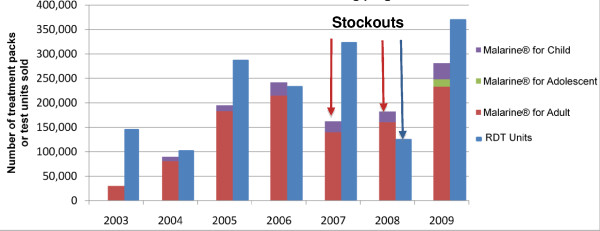
**PSI's annual sales of Malarine and Malacheck^® ^since the start of the programme**. This Figure shows the evolution of PSI's annual sales volumes for Malarine and Malacheck^® ^since the start of the nationwide programme.

Data on retail sales to end-users are not currently available. However, an overview of PSI's wholesale sales records suggests that although the general trend has been up, the sales of ACT and RDTs have fluctuated markedly both in absolute terms and in relation to each other, particularly between 2006 and 2008 (Figure 3). This is likely to be due to supply bottlenecks related to procurement problems and in this context the fluctuation of sales volumes cannot be interpreted as reflecting changes in consumer demand. In 2009, seven years after the start of the programme, wholesale sales of Malarine across all age groups and Malacheck reached their highest levels. The larger number of adult doses sold compared to child and adolescent doses reflects the relatively higher disease burden that falls on adults. Overall, PSI's sales of Malarine exceeded the number of doses of "A+M" provided through public health facilities and through VMWs[15]. However, as there are no data linking Malarine sales volumes to its use or to results of diagnostic testing it is not possible to say what proportion of the doses sold in the private sector were "appropriate".

### Discussion and evaluation

To date, there has been no formal evaluation of the social marketing programme. However, there have been a number of studies on the provision and utilization of blood testing and anti-malarials in Cambodia (Additional File 2): six surveys provided data on providers' knowledge of RDTs and ACT, product availability, sales and/or selling prices and five others on consumers' awareness of RDTs and ACT, access to confirmed diagnosis and quality treatment and costs. A brief overview of the evolution of key quantitative outcomes of the social marketing programme in terms of ACT and RDTs awareness, availability, price and use is provided in two accompanying files (Additional File 3 and Additional File 4).

Whilst these quantitative studies are not all nationally representative and differ in terms of sample sizes, methodologies and geographical areas covered (Additional File 5), they provide valuable insights into the "Cambodian experience" in improving RDTs and ACT awareness, availability, access, prices, quality and patient adherence.

### Drugs

#### Awareness of the recommended ACT

Data on awareness of recommended ACT were available from five household and/or provider surveys. During the first year of scale-up, a quarter (24%) of respondents had reportedly heard of Malarine, of which 79% said they had heard about it from the television [39]. During the same survey, around a quarter of private providers reportedly recommended to their clients the nationally recommended blister-packed ACT, that is either Malarine or the public sector "A+M" [39]. Two years later, awareness rates were higher: of 3,363 households and 123 providers surveyed, 47% and 75% respectively reported that they had heard of either Malarine or A+M [40]. By 2007, awareness had risen further to 72% of surveyed households and 98% of surveyed providers respectively for Malarine alone [41]. In the same year, however, a smaller survey found that only 79% of 750 private providers had reportedly known of Malarine, and 63% reported it to be the most effective treatment for *P. falciparum *malaria [42]. More recently, of 180 private providers surveyed in Western Cambodia, nearly all (98%) reported they had heard of Malarine [43].

#### Availability of the recommended ACT

Several provider surveys measured the relative availability of the recommended ACT compared to artemisinin monotherapy (AMT) and other drugs in private commercial outlets. In 2002, the first year of scale-up, unsurprisingly, ACT availability was low, especially in more remote areas, with none of the 107 village shops surveyed stocking Malarine and only 40% of the 49 market-based providers doing so [39]. Public sector "A+M" was available in around 30% of market-based providers and 8% of village outlets, suggesting leakage of the drugs from the public sector [39]. Artesunate monotherapies, on the other hand, were more widely available in as many as 85% of market-based providers and 70% of village shops[39]. Quinine, both in oral and injectable forms, and chloroquine tablets were also widely stocked [39]. In 2004, availability of the recommended ACT remained low: of 123 private providers interviewed, only 22% reported stocking Malarine for Adult and only 5% Malarine for Child. Public sector "A+M" was still available at 15% of private outlets and, as in 2002, AMT, quinine and chloroquine continued to be the most widely available anti-malarial drugs, and were stocked by 45%, 48% and 57% of all providers respectively [40].

By 2007, availability had increased, although the two surveys conducted in this year provide different estimates. In a survey conducted by PSI based on LQAS methodology, only about half of communes in high-risk areas had Malarine available with only 44% and 17% of the 249 providers surveyed reported generally stocking Malarine Adult and Malarine Child, respectively [44]. Penetration rates were particularly low for mobile providers, a popular source of treatment in rural areas. PSI was actually experiencing stock outs of Malarine and, without surprise, the majority of private providers reported at least one day without any Malarine in stock during the three months preceding the survey. Artesunate continued to be stocked in 19% of outlets [44]. On the other hand, the Cambodia national survey reported better availability with around 63% of the 131 providers interviewed reporting that they 'usually' stocked Malarine for Adult and 44% Malarine for Child [41]. Public sector "A+M" availability seemed to have decreased, with less than 8% of providers reporting generally stocking it [41]. However, in this study, AMT remained widely available with 41% of providers reporting they commonly stocked artesunate tablets and 18% artesunate injection[41].

By 2009 Malarine was found to be the most frequently stocked anti-malarial treatment [43]. However, the popularity of AMTs amongst some providers still prevailed, despite having been banned a year before and of 180 private providers interviewed, 58% stocked artemisinin-based drugs, including artesunate tablets and injectable artesunate and artemether [43].

#### Uptake of the recommended ACT

**Figure 4:**
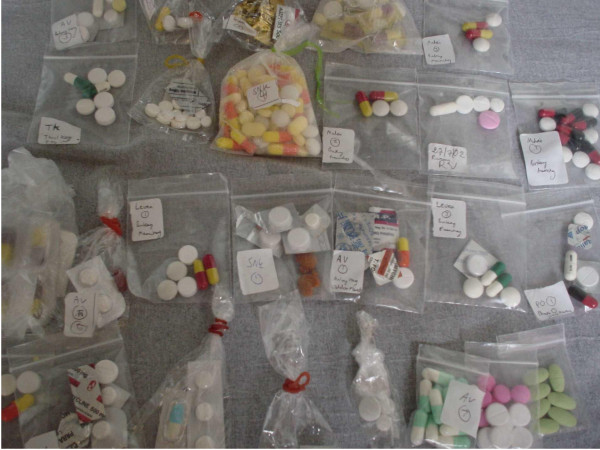
**Drug "cocktails"**. An assortment of mixed drug "cocktails", which are a mix of several different types of drugs sold in little plastic bags, often used for treating fever in Cambodia [28].

In this paper, "uptake" refers to providers' reported selling practices and households reported buying practices for recommended blister-packed ACT and other anti-malarial drugs. Before the nationwide scale-up in 2002, 361 household respondents who were interviewed on anti-malarial drug use reported the widespread use of "cocktails", which usually consisted of a mix of three or more types of tablets or capsules sold in little plastic bags [28] (Figure 4). By contrast, only 8% reported receiving the recommended blister-packed ACT [28].

Overall, 40% of all anti-malarial treatments received, or 78% of those that contained artemisinin, contained an artemisinin derivative as a monotherapy[28]. The results were corroborated by a larger study later that year, in which out of 1,277 household respondents, blister packed ASMQ accounted for less than 10% of all treatments received and the most commonly received treatments included AMTs and quinine [39]. Given the low levels of availability particularly amongst rural providers, it is not surprising that uptake remained low for several years with the use of inappropriate treatment prevailing. In 2004, the most commonly received treatment was reported to be either "drug cocktails" or "unknown"[40] and, in 2006, of 150 respondents who received treatment, only 13% reported receiving Malarine [36]. This was corroborated one year later [42,44] with, for example, only 28% of 750 providers who reported they frequently provided prepackaged ACT to patients with *P. falciparum *malaria [42]. More recent data from studies including those conducted by the ACTwatch Group and the ACT Consortium suggest that uptake has improved since then.

#### Parasitological diagnosis and RDTs

Compared to treatment, there is, unfortunately, much less information on awareness, perceptions and use of RDTs. The available data are summarized and discussed below.

#### Awareness and perception of RDTs

Before 2006, there is limited evidence on awareness of RDTs or reported perceptions towards diagnosis. In 2006, a study conducted by PSI, which used a Lickert scale to elicit communities' perceptions, reported that blood testing was perceived to be a common practice if malaria infection was suspected and that diagnosis blood tests in general were neither considered a 'waste or money' nor a 'waste of time' [36]. Also, nearly 70% of respondents reported that malaria could not be diagnosed without a blood test, although only around half perceived RDTs to be as reliable as microscopes[36]. On the providers' side, nearly 88% of respondents knew that malaria symptoms could be associated with other diseases but around a third reported that malaria infection could be confirmed without a blood test [42].

#### Availability of RDTs

In 2002, biological testing was found to be rarely available. Of 156 private providers, microscopy was reportedly offered by 18% of market-based outlets and 21% of village outlets and only 14% and 7% reportedly stocked RDTs respectively [39]. This situation appears to have remained unchanged for a number of years, with just over 10% of outlets reportedly stocking Malacheck in 2004 and just under 15% the public-sector version, Paracheck^® ^[40], suggesting some leakage. By 2007, Malacheck availability was reported to have increased [41,44]: of 131 private providers surveyed, nearly half (49%) reportedly stocked it, whilst 15% continued to stock Paracheck^® ^[41]. Overall, the pattern of Malacheck availability was noted to be similar to that of Malarine^®^, with most pharmacies, cabinets and drug stores selling the product, but less so by mobile providers [44].

#### Use of RDTs

In the community studies on anti-malarial drug usage conducted in 2002, only around 18% of patients with recent malaria symptoms reported receiving a biological diagnosis with a minority (15%) of tests having been conducted using RDT[28]. Unsurprisingly, given the lack of increase in availability, there was little change over the next few years: in 2004, less than 15% of households who had sought care for malaria symptoms reportedly received a blood test [40]. In 2007, this proportion remained only 21%, although the proportion being tested by RDT had reportedly increased to 61% [41]. These estimates were somewhat in contrast with another household study during 2006 in which 71% of those who had symptoms and sought care in the last 12 months reportedly received a diagnostic test. Out of the 109 respondents who reported having a test, two-thirds were reportedly by RDT and nearly all (98%) were reportedly positive, suggesting either inaccuracies in interpreting the test or significant bias in the responses [36]. On the supply side, of 110 providers interviewed, 85% reported they planned to provide a diagnostic test to the next patient with malaria symptoms and a similar proportion reported that they provided a test to their last malaria patient. However, only 10% of providers who reported they had treated malaria in the previous six months indicated that they always provided a diagnosis [36].

It would be of interest to explore the relationship between the use of RDTs, the result of the tests and the sale of ACT. Unfortunately, the available data do not allow for such analysis. However, some observations on the sales of RDTs relative to that of ACT can be made from the overall sales data presented in Figure 4. Although the ratio of sales has varied from year to year, the ratio has overall been close to one-to-one, except in 2007 when there was an acute shortage Malarine. PSI are currently collecting and re-reading used RDTs from private providers and preliminary findings suggest a positivity rate of around 30%, similar to that reported by village malaria workers (personal communication, PSI Cambodia malaria programme). The relatively high sales of Malarine relative to Malacheck support the observation that it is not uncommon for ACT to be sold without biological confirmation. However the difference may also be in part because of private providers confirming infection through microscopy rather than RDTs, as found recently in Western Cambodia, where of the private providers who reportedly tested patients' blood before selling anti-malarials 54% reported using microscopy only, 37% either microscopy or RDTs and 19% RDTs only [43].

### Cost and adherence to recommended pricing

Since the start of the programme, there have been recommended wholesale and retail prices for each product. However the available evidence suggests that adherence to these recommendations has been variable. During the first year of scale-up, households who had bought pre-packed ACT at private outlets reported buying the drugs at close to the recommended price of 7,900 riel, with a median of 8,000 riel (US$2). This compared to a median of 6,000 riel (US$1.46) for other anti-malarials [39]. In 2004, when the RRP for Malarine was still 7,900 riel, the national malaria survey reported that the median price paid by consumers for Malarine had reportedly fallen dramatically to 3,000 riel (US$0.73) [40]. In the same survey repeated in 2007, by which time the RRP had been reduced to US$ 0.61 [44], the median price paid was still 3,500 riel (US$0.85) [41]. However, results from a PSI study that same year, reported much higher prices being paid: Malarine for Adult, at a mean price of US$1.07 (range US$0.63-US$3.75) and Malarine for Child at US$0.95 (range US$0.63-US$2.50). This situation may however not come as a surprise in the context of supply shortages due to procurement difficulties. Furthermore, at the time retailers reportedly purchased Malarine at a mean price of around US$7.50 (range US$5.00-US$20.00) for 12 packs, well above the PSI's wholesale recommended price of $5.50 for 12 packs [44], and relatively close to the RRP, therefore, squeezing retailers' expected margins. This situation shows the important influence that the distribution chain is likely to have on retail outcomes, and highlights the limited information available on providers' business practices and pricing behaviours [45].

Similarly, in 2007, Malacheck was reported to be sold to end-users at an average price of US$0.35 (range US$0.25-US$1.25), which was US$0.10 higher than PSI's RRP (US$0.25), with price adherence being apparently lower in areas with the highest risk of malaria transmission [44]. As for the drugs, retailers reported paying on average US$2.90 (range US$1.90 - US$12.5) for 10 tests, which was US$0.70 above the wholesale recommended price of US$2.20[44].

In 2009, Malacheck was reportedly being sold by retailers for around US$0.50, or US$0.26 above the RRP[43].

### Quality and adherence

Both the drugs and the RDTs that are procured for the social marketing programme are quality assured products and undergo quality testing prior to distribution. However, there is little information available on the quality of the products after distribution or on how well they are used. The 2007 MAP survey suggested that the quality of coverage, as measured by the presence of expired stocks and correct storage conditions was generally very good [44]. However, it also found that almost one third of private providers had sold Malarine tablets individually by either removing or cutting tablets from the blister pack. More recently, about18% of surveyed outlets reportedly stocked expired anti-malarials[43]. There are no data on the quality of RDTs - neither regarding the quality of the tests themselves after they have been stored under normal conditions, nor on how well they are used in practice.

There is also little information on patients' adherence in terms of completion of recommended treatment courses. The studies of anti-malarial drug use in 2002 assessed adherence based on the self-reported duration of treatment with different anti-malarials. In all, 41.1% (95% CI 36.4, 45.7) of respondents had received anti-malarials for inadequate treatment duration. For the recommended pre-packaged ACT adherence was relatively better with 72-78% of patients who took it reportedly taking the treatment for the recommended 3 days [28,39] although only 62% of patients reported completing the whole package [28]. Adherence was lowest with treatments that included quinine, mefloquine, chloroquine and artemisinin monotherapies. When artesunate was taken alone and not as part of a pre-packaged treatment, only 13-28% took it for the recommended duration of 7 days[28]. By 2006, around 71% of 675 household respondents reportedly knew that pre-packed malaria treatment was effective only if the entire course was taken [36].

## Conclusions

With the imminent roll-out of the AMFm pilot in selected malaria-endemic countries, there has been much discussion about its likely impact on the availability, uptake and end-user price of ACT in the private sector [46-48] and the potential role of RDTs [49,50]. ACT subsidy schemes have already been implemented in a limited number of countries, generally at a smaller scale (e.g district) [12,14,51-55]. These suggest that a subsidy on ACT can rapidly increase ACT availability in private outlets, decrease ACT consumer prices, increase ACT uptake and decrease artemisinin monotherapy use, although these effects may tend to benefit relatively accessible populations rather than the more remote and poorer communities. Whilst providing useful insights, most of these initiatives have been pilot studies, whose implementation benefited from intense support and monitoring activities, and which were limited to ACT only. Therefore to date there is no detailed information on the outcomes of subsidy schemes for both ACT and RDT that have been implemented at national level and that could guide the scale-up of ACT and introduction of RDTs.

Cambodia is the first and only country with a mature nation-wide social marketing programme, not only of subsidised ACT, but also of RDTs. In the absence of a formal evaluation, this case study has drawn together the available data on the impact of this national programme. The approach taken has inherent limitations due the variability between studies in terms of design, geographical areas covered and measured indicators. However, useful lessons can be learnt for the AMFm and the implementation and evaluation of similar nationwide programmes of subsidised ACT and RDTs in the private sector (Table 1).

**Table 1:** Key lessons and implications for the implementation and evaluation of nationwide programmes of subsidised ACT and RDTs in the private sector.

Programme evaluation	
**Lessons learnt**	**Implications**
• Difficult to assess the impact of programme due to the lack of routine monitoring with standardised indicators, especially at the household level	• Need for routine monitoring and evaluation using standardised indicators to measure impact on specific programme objectives, particularly in more remote areas:
• Could not assess ACT uptake, *appropriateness *of ACT uptake, *quality *of ACT and RDTs and *how *these products are used	• At the household level (in addition to the standard indicator of access to prompt treatment of fevers)
	○ Access to affordable, good quality parasitological diagnosis prior to treatment with ACT ○ Adherence to ACT treatment regimen ○ Use of sub-standard drugs, in particular sub-standard artemisinin drugs and artemisinin monotherapies ○ Median price paid for recommended ACT (compared to the most popular anti-malarial)
	• At drug outlet level (in addition to indicators of awareness, availability and price)
	○ Quality of RDTs and ACT under field conditions ○ Correct use and interpretation of RDTs by providers ○ Correct treatment regimen dispensed by providers
• Difficult to assess *why *things worked or did not work	• Consider a comprehensive evaluation of the implementation process using qualitative methods, in order to understand how implementation can and should be improved
**Programme implementation**	
**Lessons learnt**	**Implications**
• A high level of brand awareness was achieved through an effective behaviour change communication strategy about Malarine • Awareness, availability and uptake of Malacheck was lower than for Malarine • Availability of both ACT and RDTs took years to pick up and was particularly low in rural areas and with mobile providers. In part this is explained by supply bottlenecks. • Despite Malarine being available, actual uptake remained low compared to other anti-malarial drugs. Problems with uptake likely to be associated with community perceptions and expectations.	• Depending on the setting, significant additional resources may be required to raise awareness and knowledge through IEC activities • In order to ensure improved targeting of ACT to patients with malaria, attention needs to be paid to biological testing in behaviour change communications, and in the training and support of private providers. This is particularly important in low transmission settings where the majority of fevers are not due to malaria • Need to monitor availability of products in remote areas and consider interventions to improve reach • Need to ensure reliable supply of products • Need to consider different strategies for different types of providers i.e. mobile providers in rural areas versus trained formal providers in market towns • Need to monitor actual use; if it remains limited, seek to understand why and address the underlying problem(s) - which may require consideration of changing the price, the product (eg switching from co-blistered mefloquine and artesunate) or/and modifying the communications strategy.

In terms of evaluation, the limitations in this case study highlight the need for a comprehensive framework for evaluation [56] and the systematic collection of standardised indicators. In view of the original aims of a high level subsidy for good-quality ACT[57], this should include household level data on access to affordable good-quality ACT and the relative availability of sub-standard drugs and artemisinin monotherapies. This is essential in order to inform timely and appropriate modification to the implementation process. With decreasing prevalence of malaria and the emergence of artemisinin resistance in Cambodia, there is also increasing urgency to monitor access to parasitological diagnosis and the appropriate use of RDTs and ACT, and to collect surveillance data from private providers.

As for programme implementation, it is evident that after 10 years of social marketing, Malarine is widely known and appears to be the most popular anti-malarial. However, this has taken many years to achieve, and there are lessons to be learnt from the process, as well as the ongoing challenges.

The first lesson concerns product awareness. In order for providers to stock and sell the recommended ACT and RDTs and to encourage consumers to buy these products, awareness of the products is clearly important. In Cambodia, there was a relatively rapid increase in brand awareness for the drug Malarine. Achieving this has required an intense and sustained behaviour change communication (BCC) strategy, which includes provider training, radio and television advertisements, mobile community video units and job aids. If similar levels of awareness are to be achieved for subsidised ACT in countries rolling out the AMFm, it is likely that similar campaigns will be required, and adequate resources made available. Awareness of the RDT, Malacheck remained lower than that of Malarine, reflecting the difference in emphasis in the marketing of the products. For other malaria-endemic countries who are rolling out both RDTs and ACT, careful consideration will need to be given to providing adequate consumer and provider information and training on the role and use of RDTs from the start of the programme.

The second lesson concerns product availability. The data suggest that it took several years for Malarine and Malacheck to become readily available, especially in rural areas and particularly through mobile providers, a popular source of treatment. They also suggest that the RRP for both products was poorly adhered to. In part these finding are explained by intermittent bottlenecks in procurement and the resulting central stock-outs. This highlights the importance of ensuring a reliable supply of goods, and the need for far-reaching distribution networks to ensure availability of effective treatment in the least accessible areas. It also suggests that different strategies may be needed to target different types of providers, for example informal providers in rural areas versus trained formal providers in market towns. Although the problems with the procurement and supply of ACT and RDTs go some way to explaining their slow uptake by providers and consumers, there are a number of other factors that have contributed. One important factor influencing the slow uptake of Malarine is the product itself. The key elements of marketing are sometimes referred to as the "4 Ps" - product, price, place and promotion. In the case of Malarine, the price seems competitive; the promotion is intense and targets both consumers and providers; and, the packaging is attractive. In terms of place, PSI distributes to a wide range of providers in market towns, relying on the existing distribution chain to reach outlets operating at the periphery. However, there have been challenges with the product itself. In Cambodia, the individual components of Malarine - artesunate and mefloquine - had been available for several years prior to their introduction as a combination therapy into national policy. There has been a common perception that mefloquine causes unpleasant side effects, whilst artesunate on its own is effective (which it is not, unless taken for 7 days) and well-tolerated. Until the recent ban on the artesunate monotherapy, there was little incentive for consumers to buy a pack of Malarine instead of a pack of artesunate. Even now, a patient can choose to buy Malarine, but to take only the artesunate tablets. How much this might contributes to the development of artemisinin resistance in Cambodia is unknown but is of great concern, and highlights the potential negative consequences of the severe delays in the introduction of co-formulated ACT in Cambodia [58].

As for the ongoing challenges in increasing uptake of RDTs, one important challenge is the complexity of the message. The social marketing of subsidised RDTs and ACT for malaria is different from that of other public health commodities, such as bed nets, condoms or contraceptives. In most of these cases, there is a simple goal - to maximize sales, and a single message - "buy this product". For RDTs and ACT, it is more complicated because there is more than one objective and more than one message. The key objectives are to maximise the sales of ACT to the specific population of patients with malaria, whilst minimising sales of anti-malarials to the non-target populations. There is therefore at least three other key messages: firstly, "if you are going to buy an anti-malarial, only buy the recommended ACT"; secondly, "before you buy an anti-malarial, get tested first"; and thirdly, "if you test negative, don't take an anti-malarial". In addition, there are a number of other important messages, including for example the importance of adherence to the recommended course and appropriate referral to the public health facilities. Where non-*falciparum *malaria is common, as in Cambodia, the message gets even more complicated.

The third lesson concerns product pricing. Although unlikely to be sufficient on their own, financial incentives can be used to help to achieve the desired provider and consumer behaviors with regard to diagnosis and treatment. In Cambodia, RDTs are now sold to retailers at almost one-tenth the cost of the adult drug (US$0.05 versus US$0.42) and should theoretically allow retailers to make a 380% gross profit (assuming they receive their supplies from PSI directly), whilst the recommended retail price of the RDT is less than half the cost of the adult drug (US$0.24 versus US$0.61). Other countries considering the promotion of RDT use in the private sector will need to consider the resources required to provide financial incentives to promote their uptake alongside sufficient training and support of private providers.

In addition it is important to identify and address other non-financial barriers to the uptake and appropriate use of RDTs. One likely contributory factor for providers' reluctance to use RDTs or trust their results is the lack of clarity about what to do when the result is negative. Although referral to a public health facility is advised, in reality this rarely takes place. There is an urgent need for simple algorithms, which guide the management of treatment in the "RDT negative" patients and better evidence on the aetiology of non-malarial febrile illness on which to base such algorithms.

Finally, it is important to be clear of the role of the private sector in malaria diagnosis and treatment in the context of the health system as a whole. In Cambodia, and in many other developing countries, the aspiration is to provide better access to good quality care through the public sector. Cornerstones of this strategy may include strengthening the provision of care at public health facilities and the provision of free diagnosis and treatment through trained village volunteers.

## Competing interests

The authors declare that they have no competing interests.

## Authors' contributions

SY conceived the case study and collated the different studies. SY and EP analysed the data and drafted the manuscript. MS, HA and DS helped in undertaking the background research and editing the manuscript. All authors read and approved the final manuscript.

## Authors' information

SY is a Clinical Senior Lecturer in the Department of Global Health and Development and Department of Clinical Research at the London School of Hygiene and Tropical Medicine. She is a vice-director and core scientist for the ACT consortium, which is supported by a grant from the Bill and Melinda Gates Foundation. EP is a Research Fellow in the Department of Global Health and Development. She is a member of the ACTwatch Group, which is supported by a grant from the Bill and Melinda Gates Foundation. MS is the PSI Cambodia Malaria Programme Officer. HA is the PSI Cambodia Malaria Programme Technical Advisor. DS is the former Director of the Cambodia National Malaria Control Programme.

## Supplementary Material

Additional file 1 **Programmatic prices and margins for PSI's socially marketed ACT and RDT**. Additional File 1 presents programmatic prices and margins for PSI's socially marketed ACT and RDT Click here for file

Additional file 2 **Overview of the reviewed literature**. Additional file 2 describes the design of large surveys that provided evidence on ACT and/or RDT awareness, availability, use and price since the start of the programme Click here for file

Additional file 3 **Overview of survey results on ACT awareness, availability and use**. Additional File 3 provides an overview of the key findings on the evolution of ACT-related outcomes since the start of the social marketing programme, in terms of ACT awareness, availability and use from both provider and household surveys. Click here for file

Additional file 4 **Overview of study findings on RDT awareness, availability and use**. Additional File 4 provides an overview of the key findings on the evolution of RDT-related outcomes since the start of the social marketing programme, in terms of RDT awareness, availability and use from both provider and household surveys. Click here for file

Additional file 5 **Map of survey sites**. The map shows the malaria-endemic provinces covered in the sampling for nationwide surveys, and the survey sites for the three studies which focused on the areas of high antimalarial drug resistance in South and Western Cambodia Click here for file
