# Supplementary material for: Socially-marketed rapid diagnostic tests and ACT in the private sector: ten years of experience in Cambodia
Source: Malar J. 2011 Aug 18;10:243. doi: 10.1186/1475-2875-10-243 (PMC3173399; doi:10.1186/1475-2875-10-243)
Supplement: Additional file 1 — Programmatic prices and margins for PSI's socially marketed ACT and RDT. Additional File 1 presents programmatic prices and margins for PSI's socially marketed ACT and RDT [file 1475-2875-10-243-S1.DOC]

|  | Before May 2009 | | | Since May 2009 | | |
| --- | --- | --- | --- | --- | --- | --- |
|  | PSI’s Selling Price to Retailers | PSI’s RRP^1^ | Distribution margin, in US$ (%)^2^ | PSI Selling Price to Retailers and Wholesalers | PSI’s RRP^1^ | Distribution margin, in US$ (%)^2^ |
| Malarine® Adult | US$ 0.46 | US$ 0.61 | US$ 0.15 (33%) | US$ 0.42 | US$ 0.61 | US$ 0.19 (45%) |
| Malarine® Child | US$ 0.46 | US$ 0.61 | US$ 0.15 (33%) | US$ 0.18 | US$ 0.29 | US$ 0.11 (61%) |
| Malarine® Adolescent^3^ | - | - | - | US$ 0.26 | US$ 0.41 | US$ 0.15 (58%) |
| Malacheck® | US$ 0.22 | US$ 0.24 | US$ 0.02 (9%) | US$ 0.05 | US$ 0.24 | US$ 0.19 (380%) |

^1^ RRP is recommended retail price; ^2^ Distribution margin is the difference between the RRP and the price paid by retailers when buying Malarine® or Malacheck® from PSI directly. In percent terms, the margin is calculated as the percentage variation between RRP and PSI’s Selling Price; ^3^Malarine® Adolescent was introduced in May 2009.
