# Supplementary material for: Socially-marketed rapid diagnostic tests and ACT in the private sector: ten years of experience in Cambodia
Source: Malar J. 2011 Aug 18;10:243. doi: 10.1186/1475-2875-10-243 (PMC3173399; doi:10.1186/1475-2875-10-243)
Supplement: Additional file 2 — Overview of the reviewed literature. Additional file 2 describes the design of large surveys that provided evidence on ACT and/or RDT awareness, availability, use and price since the start of the programme [file 1475-2875-10-243-S2.DOC]

| **Year of survey** | **2002** | **2002** | **2004** | **2006** | **2007** | **2007** | **2007** | **2009** |
| --- | --- | --- | --- | --- | --- | --- | --- | --- |
| **Month** | **July-Sept** | **October** | **November-December** | **June-July** | **September** | **October** | **December** | **July** |
| **Study Reference** | **Yeung[28]** | **CDUS[39]** | **CMBS[40]** | **PSI Household TRaC^1^[36]** | **PSI MAP 2007[44]** | **PSI Provider TRAC[42]** | **CMS 2007[41]** | **URC-MC[43]** |
| **Study type** | Household cross-sectional survey | Household & Provider cross-sectional surveys | Household & Provider cross-sectional surveys | Household cross-sectional survey | Provider cross-sectional survey | Provider cross-sectional survey | Household & Provider cross-sectional surveys | Provider cross-sectional survey |
| **Sample selection** | •Sample frame: all villages in 3 “intervention” areas^2^: districts with VMW, with outreach clinics and without specific intervention; Villages stratified into 2 groups depending on accessibility  • Sampling procedure: random selection of villages ^3^, then visit all households and screen for recent fever cases. | •Sample frame: all villages in 4 districts with drug sensitivity trials and other 5 districts randomly selected from neighbouring areas^4^; Villages stratified by distance to health centre  •Sampling procedure: random selection of villages, then visit all households and all public & convenient sampling of market based and village based private providers | •Sample frame: all endemic villages classified into 3 malaria transmission risk zones: high, medium and low ^5^ and provinces classified into 3 domains (according to forest type)  •Sampling procedure: random selection of villages in each domain, then random selection of 40 households in each village, & all or up to 4 household members interviewed^6^; for the provider survey, convenient sampling of private providers | •Sample frame: in 17 endemic provinces, all villages stratified in high, medium and low transmission risk zones^5^  •Sampling procedure: in each stratum, villages selected by probability proportional to size and in each village, random sample of households | •Sample frame: all communes in high, medium and low risk zones  •Sampling procedure: random sample of 19 communes,  (LQAS^7^), then visit all health outlets stocking PSI socially marketed RDT and ACT | •Sample frame: all 17 endemic provinces  •Sampling procedure: random sample of 6 provinces using PPS^7^, then stratification of all communes in high, medium and low transmission risk zone; from each stratum, random sample of 90 communes by PPS, then interview up to 4 randomly selected providers. | •Sample frame: CMBS 2004 sample frame, with one additional risk zone^5^ and only 2 province categories/ domain^9^  •Sampling procedure: same as CMBS 2004 | •Sample frame: used existing lists of private providers (available from PSI and district authorities) in 5 operational districts  •Sampling procedures: convenient sampling of private providers |
| **Sample size** | •361 household respondents | •1277 household respondents  •49 market based - providers and 107 village shops | •3363 household respondents  •43 market –based providers and 80 village shops | •675 households respondents | •110 private health providers^8^ | •750 private providers^8^ | •2924 households respondents  •131 private providers | •180 private providers |
| **^1^**TRaC is a PSI study type Tracking Result Continuously; ^2^ District with Village Malaria Workers, 1 with outreach clinic and 4 with no intervention; all villages are those covered by the district public health facilities and with reported malaria cases; ^3^ 14 ‘control’ villages, 7 with outreach clinics and 2 with village malaria workers; villages across 3 clusters having similar characteristics in terms of malaria transmission risk, ecology, access to road, health centres and markets, and communities’ socio-economic status, poverty and level of migration were selected; ^4^selected for comparative purposes with follow-up research activities on drug use and drug resistance level; ^5^ CMBS 2004: high risk transmission zone includes villages located in or less than 250 metres from the forest; low risk zone villages located between 250 metres and 1 kilometre from forest and low risk zone includes villages located between 1 and 2 kilometres away from the forest; PSI TRAC 2006: high risk villages are those located less than 1 kilometre from the forest; medium risk villages within 1 to 2 kilometres of the forest and low risk villages more than 2 kilometres from the forest; CMS 2007: a risk zone including villages within 2 to 5 kilometres from the forest was added to the 3 zones used at baseline during the 2004 CMBS; ^6^ 1 adult female, 1 adult male, 1 infant aged 0-4 years old and 1 child 5-14 years old; ^7^ LQAS is Lot Quality Assurance Sampling procedure; PPS is Probability Proportional to Size; ^8^ MAP 2007: private health providers included those working at pharmacies, clinical pharmacies, cabinets, drug shops and mobile providers; in the TRAC 2007,providers are qualified, semi-qualified and non-qualified providers; qualified private providers included medical doctors and assistants, and pharmacists; semi-qualified private providers included nurses, midwives, pharmaceutical assistants, and laboratory technicians; non-qualified private providers included informal drug shopkeepers;^9^ one province category/domain dropped because of extremely low malaria prevalence | | | | | | | | |
