# Supplementary material for: Socially-marketed rapid diagnostic tests and ACT in the private sector: ten years of experience in Cambodia
Source: Malar J. 2011 Aug 18;10:243. doi: 10.1186/1475-2875-10-243 (PMC3173399; doi:10.1186/1475-2875-10-243)
Supplement: Additional file 3 — Overview of survey results on ACT awareness, availability and use. Additional File 3 provides an overview of the key findings on the evolution of ACT-related outcomes since the start of the social marketing programme, in terms of ACT awareness, availability and use from both provider and household surveys. [file 1475-2875-10-243-S3.DOC]

| **Year of survey** | **2002** | **2002** | **2004** | **2006** | **2007** | **2007** | **2007** | **2009** |
| --- | --- | --- | --- | --- | --- | --- | --- | --- |
| **Survey Name^1^ and study population** | **Yeung et al. [28]**  **Households** | **CDUS [39]**  **Households & Providers** | **CMBS [40]**  **Households & Providers** | **TraC [36]**  **Households** | **MAP [44]**  **Providers** | **TraC [42]**  **Providers** | **CMS [41]**  **Households & Providers** | **URC-MCC [43]**  **Providers** |
| **AWARENESS of first-line pre-packaged treatment artemisinin combination Therapy (PPT ACT)**^3^ | | | | | | | | |
| **Providers** |  | •22-25% of providers mentioned Malarine or A+M as the recommended 1^st^ line treatment | •75% of providers reported they had heard of either Malarine or A+M |  |  | •78.5% of providers reported they had heard of Malarine® | •98% of providers reported they had heard of either Malarine or A+M | •98% of private providers reported they had heard of Malarine®, 64% Artekin (DHA+PP) and 87% A+M  •44% reported that Malarine is popularly known |
| **Households (HH)** |  | •24% of HH respondents reported they had heard about Malarine | •47% (95%CI; 38.3-56.4) of HH respondents reported they had heard of either Malarine or A+M | •79.6% of HH respondents reported they had heard of Malarine® |  |  | •72% (95% CI: 66.4-76.9) of HH respondents reported they had heard of either Malarine or A+M |  |
| **AVAILABILITY of PPT ACT^2^  and artemisinin monotherapies** | | | | | | | | |
| **PPT ACT** |  | • None of the village providers 40% of market providers and stocked Malarine®.  • 8% of village providers and more than 30% of market providers stocked public sector A+M. | •22% of providers^4^ stocked Malarine adult and 4.9% Malarine ®child.  •14.6% of private providers stocked A+M adult dose and 7.3% A+M children doses |  | •43.8% of providers^4^ stocked Malarine adult and 17.3% Malarine child  •5.2% stocked public sector A+M adult and 3.6% public sector A+M children doses |  | •62.6% of providers reported usually stocking Malarine adult and 44.3% Malarine child  •7.6% reported they usually stocked A+M | •67% of providers who had heard of Malarine stocked it  •36% of private providers who had heard of Artekin stocked it  •5%of private providers who had heard of A+M stocked it  •Most frequently stocked antimalarial was Malarine adult |
| **Artemisinin monotherapies**^3^ |  | •85% of market providers and 70% of village providers stocked AS tablets^5^ | •44.7% of providers stocked AS tablet, 25.2% AS injection and 19.5% AR^5^ |  | •18.8% of all drugs in stock that were used for malaria treatment were AS  •45.2% of providers selling cocktail drugs to treat malaria reported that cocktails contained AS |  | •41.2% of providers stocked AS tablets, 18.3% AS injection | •58% of private providers stocked AMT  •48.7% of private providers had AS tablet in stock, 25.3% AS injection and 33.5% AR injection |
| **USE** | | | | | | | | |
| **Providers reported selling practises** |  | •50% of market providers and 11% of village providers reported that they would recommend ASMQ for treating uncomplicated PF in adult |  |  |  | •28.3% of providers reported frequently providing PPT ACT |  |  |
| **Households reported use** | •8% (17/210) of respondents who used modern drugs received PPT ACT  • 40% of all antimalarial treatments contained of AS  (See text for information on adherence) | • 7% of HH respondents who received an antimalarial reported receiving PPT ACT, with 4% receiving Malarine and 3% A+M  •3% of all modern treatment received were Malarine®, 6% were A+M  •32% (95% CI 17.2-46.5) of HH respondents who received public sector version A+M reported they received it from a private provider |  | •13.3% of HH respondents who received treatment (n=150) reported they received Malarine |  |  |  |  |
| **PRICE** | | | | | | | | |
| **•Providers reported selling price** |  |  | •US$0.73 is median selling price for Malarine |  | • US$ 1.07 and US$ 0.95 are mean selling prices for Malarine adult & child |  | •US$0.85 is median selling price for Malarine | •US$0.88 is mean selling price for Malarine adult and US$0.83 for Malarine child |
| **•Households reported buying price** | •US$0.77 is median paid for PPT ACT | •US$2.00 is median cost paid for PPT ACT |  | •US$1.34 is average cost paid for Malarine adult |  |  |  |  |

^1^ CDUS is for Community Drug Use practice Survey; CMBS is for Community Malaria Baseline Survey; TraC is for Tracking Result Continuously study; MAP is for Measuring Access and Performance study; CMS is for Cambodia Malaria Survey; URC-MCC is for University Research Consortium-Malaria Control in Cambodia study;

^2^ Availability is defined as outlet stocking on day of survey;

^3^ First-line pre-packaged (PPT) ACT is pre-packed combination of artesunate and mefloquine, branded Malarine in the private sector and referred to as A+M in the public sector;

^4^ In the PSI MAP, providers were health outlets that included pharmacies, clinical pharmacies, cabinet, drug shops and mobile providers; in the CMBS 2004, providers were health and non-health outlets including clinics, pharmacies, drug shops, market-based provider and general shops;

^5^ AMT is artemisinin monotherapies; AS is artesunate; DHA+PP is dihydroartemisinin + piperaquine; AR is artemether
