# Supplementary material for: Socially-marketed rapid diagnostic tests and ACT in the private sector: ten years of experience in Cambodia
Source: Malar J. 2011 Aug 18;10:243. doi: 10.1186/1475-2875-10-243 (PMC3173399; doi:10.1186/1475-2875-10-243)
Supplement: Additional file 4 — Overview of study findings on RDT awareness, availability and use. Additional File 4 provides an overview of the key findings on the evolution of RDT-related outcomes since the start of the social marketing programme, in terms of RDT awareness, availability and use from both provider and household surveys. [file 1475-2875-10-243-S4.DOC]

| **Year of survey** | **2002** | **2002** | **2004** | **2006** | **2007** | **2007** | **2007** | **2009** |
| --- | --- | --- | --- | --- | --- | --- | --- | --- |
| **Survey Name^1^**  **and study population** | **Yeung et al. [29]**  **Households** | **CDUS [39]**  **Households & Providers** | **CMBS [40]**  **Households & Providers** | **TraC [36]**  **Households** | **MAP [44]**  **Providers** | **TraC [42]**  **Providers** | **CMS [41]**  **Households & Providers** | **URC-MCC [43]**  **Providers** |
| **AWARENESS of diagnosis and Rapid Diagnostic Tests (RDTs)** | | | | | | | | |
| **Providers** |  |  |  |  |  | •51.5% of providers reported they knew how to use Malacheck |  |  |
| **Households** |  |  |  | •6.4% of HH respondents reported they had heard of Malacheck |  |  |  |  |
| **AVAILABILITY^2^ of diagnosis and Rapid Diagnostic Tests (RDTs)** | | | | | | | | |
| **RDT and microscopy services** |  | •31% of village providers and 36% of market providers and reportedly provided parasitological diagnosis  •7% of village and 14% of market providers reportedly stocked RDT  •21% of village providers and 18% of market providers reportedly having microscopy services | •10% of providers stocked Malacheck  •14.6% of providers stocked the public sector Paracheck® |  | •41.8% of providers (health outlets) stocked Malacheck®  •4.8% of providers stocked public sector Paracheck®  •8.8% of providers offered microscopy services |  | •48.8% of providers stocked Malacheck  •14.5% of providers stocked the public sector Paracheck® |  |
| **USE of diagnosis and Rapid Diagnostic Tests (RDTs)** | | | | | | | | |
| **Providers reported selling practises** |  | •21%-36% reportedly said they would treat suspected malaria in children as malaria without confirmation with blood test |  |  |  | •10.1% of providers who had treated malaria in the  past 6 months reportedly said they always performed a test  •85% of providers reportedly provided a test to their last malaria patients | •37.4% of providers reportedly sold RDT in the past 3 months  •51.2% of providers reportedly sold  neither RDT nor microscopy services | •91% of private providers reportedly tested patients’ blood before providing treatment  •37% of providers reportedly used RDT or a microscope, 19% RDT only and 54% microscope only |
| **Households reported access** | •17% of HH respondents in non-intervention areas reportedly had a blood test at most recent visit  •18% of HH respondents who sought care in private sector in non-interventions areas reportedly had a blood test and 15% of tests were by RDT. |  | •14.5% of HH respondents reportedly had a blood test | •26.9% of HH respondents who ever took a blood tests reportedly said they always take a test when having symptoms of malaria  •71.1% of HH respondents who reportedly had symptoms and sought treatment in last 12 months had a blood test |  |  | •21% of HH respondents reportedly had a blood test, of which 61.2% reportedly by a RDT |  |
| **PRICE of diagnosis and Rapid Diagnostic Tests (RDTs)** | | | | | | | | |
| **Providers reported selling price** |  |  |  |  | •Mean reported selling price of one Malacheck test was US$ 0.35 (range US$0.25-US$1.25) |  |  | • Mean reported selling price of one Malacheck test was US$0.51 |

^1^ CDUS is for Community Drug Use practice Survey; CMBS is for Community Malaria Baseline Survey; Trac is for Tracking Result Continuously study; MAP is for Measuring Access and Performance study; CMS is for Cambodia Malaria Survey; URC-MCC is for University Research Co-Malaria Control in Cambodia study;

^2^ Availability is defined as outlet stocking.
