# Supplementary figures and images for: Socially-marketed rapid diagnostic tests and ACT in the private sector: ten years of experience in Cambodia
Source: Malar J. 2011 Aug 18;10:243. doi: 10.1186/1475-2875-10-243 (PMC3173399; doi:10.1186/1475-2875-10-243)

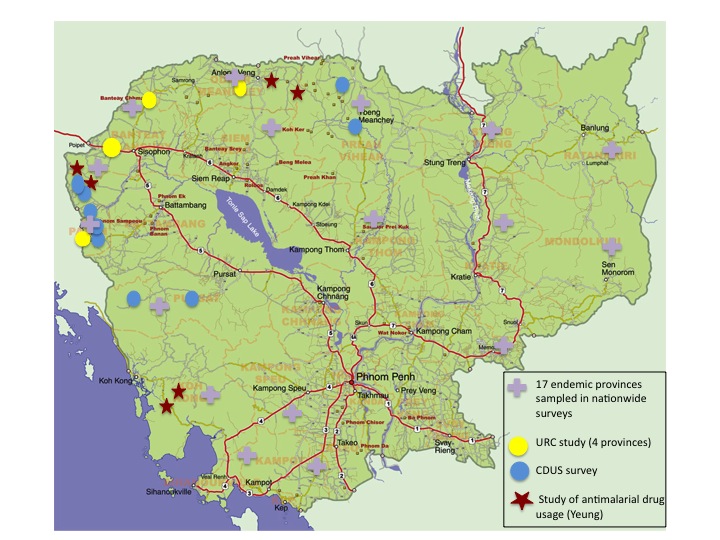

Supplement: Additional file 5 — Map of survey sites. The map shows the malaria-endemic provinces covered in the sampling for nationwide surveys, and the survey sites for the three studies which focused on the areas of high antimalarial drug resistance in South and Western Cambodia [file 1475-2875-10-243-S5.JPEG]
